# Biodistribution Study of Niosomes in Tumor-Implanted BALB/C Mice Using Scintigraphic Imaging

**DOI:** 10.3389/fphar.2021.778396

**Published:** 2022-01-07

**Authors:** Leanne De Silva, Ju-Yen Fu, Thet Thet Htar, Wan Hamirul Bahrin Wan Kamal, Azahari Kasbollah, Saravanan Muniyandy, Lay-Hong Chuah

**Affiliations:** ^1^ School of Pharmacy, Monash University Malaysia, Bandar Sunway, Malaysia; ^2^ Nutrition Unit, Malaysian Palm Oil Board, Bandar Baru Bangi, Malaysia; ^3^ Medical Technology Division, Malaysian Nuclear Agency, Bangi, Malaysia; ^4^ Department of Pharmacy, Fatima College of Health Sciences, Al Ain, United Arab Emirates

**Keywords:** biodistribution, nanotechnology, nanocarrier, niosomes, radiolabeling, gamma scintigraphy

## Abstract

The purpose of this work was to study the biodistribution of niosomes in tumor-implanted BALB/c mice using gamma scintigraphy. Niosomes were first formulated and characterized, then radiolabeled with Technetium-99 m (^99m^Tc). The biodistribution of 99mTc-labeled niosomes was evaluated in tumor-bearing mice through intravenous injection and imaged with gamma scintigraphy. The labeled complexes possessed high radiolabeling efficiency (98.08%) and were stable *in vitro* (>80% after 8 h). Scintigraphic imaging showed negligible accumulation in the stomach and thyroid, indicating minimal leaching of the radiolabel *in vivo.* Radioactivity was found mainly in the liver, spleen and kidneys. Tumor-to-muscle ratio indicated a higher specificity of the formulation for the tumor area. Overall, the formulated niosomes are stable both *in vitro* and *in vivo*, and show preferential tumor accumulation.

## 1 Introduction

There has been a rapid growth of interest in the field of nanocarriers particularly for its application in cancer therapy in terms of imaging, diagnosis and treatment over the past 3 decades (Hafner et al., 2014; Choudhury et al., 2018; Gao et al., 2018; Hussain et al., 2021). Some commonly researched nanocarriers include polymeric nanospheres, polymeric micelles, dendrimers, gold nanoparticles, iron oxide nanoparticles, solid lipid carriers and vesicular systems such a liposomes, transferomes and niosomes. To date, there are close to 250 nanomedical products which have either been approved or are currently in the final stages of approval by the Food and Drug Administration (FDA) and European Medicines Agency (EMA) for the treatment of a variety of diseases including cancer (Etheridge et al., 2013; Goldman et al., 2017). Currently, the most widely used anti-cancer nanoformulations in the market are for breast cancer treatment sold under the tradenames, Doxil and Abraxane.

The strategy of developing a magic bullet drug against a wide range of solid tumors would be an impossible task due to the vast genetic diversification and mutations of cancer in different individuals (Björnmalm et al., 2017). The enhanced permeation and retention (EPR) effect was widely used in the development of nanocarriers for passive targeting (Subhan et al., 2021), where it involves the preferential accumulation of nanocarriers within solid tumors due to their leaky vessels that are formed as a result of uncontrolled angiogenesis (Baban et al., 1998; Byrne et al., 2008; Hatakeyama et al., 2011). Therefore, this brings about the postulation that systemically administered nanocarriers that are smaller in diameter than the fenestrations, but larger than the tight endothelial junctions, have the potential to gain access within the interstitium and be entrapped within the tumor microenvironment (He et al., 2020; Shi et al., 2020). Furthermore, this accumulation is further facilitated by the tumor’s lack of well-defined lymphatic vessels that impairs extravasation of the nanocarrier out of the tumor (Haley et al., 2008; Hak et al., 2010).

Incomplete understanding on the nature of EPR has led to extensive debate in recent years. However, Maeda et al. (2021) suggested that the success or failure of EPR is highly dependent on the design of nanomedicine rather than the EPR itself. An important prerequisite for a nanocarrier is a reasonably long enough half-life to enable the EPR effect to take place. Additionally, another obstacle to effective drug delivery to the tumor microenvironment is the clearance by the mononuclear phagocyte system (MPS) (Allen and Cullis, 2013; Chapman et al., 2013). Upon entering the circulatory system, nanocarriers are often opsonized and taken up by phagocytic cells in the blood, spleen, liver and bone marrow, leaving only a small fraction of nanocarriers in the circulation that would be available for uptake at the tumor site (Chapman et al., 2013). This barrier was first overcome in the early 1990s, by another major breakthrough in the field of drug delivery with the invention of long circulating liposomes that was achieved by incorporating poly (ethylene glycol) (PEG) into the bilayer (Hatakeyama et al., 2011). This theory arose from the observation that hydrophobic particles tend to be opsonized more rapidly in the bloodstream due to the increased adsorption of blood serum proteins on their surfaces. Studies have since shown that the presence of PEG on the surface of nanoparticles can increase its blood circulation time. This is attributed to the increase in hydrophilicity, which enhances the stability of the bilayer while simultaneously introducing steric hindrance, resulting in decreased recognition by the MPS and inhibition of opsonization through protein adsorption (Hussain et al., 2019). Taken together, this results in prolonged biodistribution of the nanocarrier in the bloodstream (Allen et al., 1991; Haley et al., 2008; Hatakeyama et al., 2011; Muthu et al., 2011; Hussain et al., 2019). Another crucial factor in enabling EPR is the size of nanocarriers. It is often suggested that a size of 50–200 nm is preferred to fit tumor vascular fenestration (Nel et al., 2017) whereas sizes above 100 nm is preferred to escape renal clearance (Maeda 2021). Although low molecular weight compounds can similarly achieve passive targeting and show selective tumor uptake due to their size range, prolonged retention in the tumor can only be seen in nanomedicine (Maeda, 2021).

Niosomes are lipid bilayer nanocarriers which have previously been shown to promote tumor selective delivery of chemotherapeutics such as tamoxifen (Shaker et al., 2015) and vinblastine (Amiri et al., 2018). Niosomes fabricated in the nanometer range has been proven to be able to encapsulate hydrophobic and hydrophilic compounds (Maniam et al., 2021). In our previous study, niosomes prepared with Span 60 were presented with high biocompatibility and stability in human serum (De Silva et al., 2019). That being said, some suggested that vesicle nanocarriers like liposomes may not release encapsulated compounds even if they are delivered to the tumors via EPR effect if the liposomes are too stable (Maeda, 2021). To date, little is understood on the biodistribution of niosomes in relation to EPR mechanisms.

Determining the fate of a nanocarrier *in vivo via* biodistribution studies is a vital component in evaluating its potential safety and efficacy. Biodistribution studies typically involve the administration of a compound followed by quantifying the amount of the compound in various tissues and organs at different time-points (Patel et al., 2016). More recently, imaging techniques has attracted a lot of attention as they are less invasive and time consuming; at the same time being more cost effective in terms of quantity of animals and analytical reagents used (Ding et al., 2012; Patel et al., 2016). An example includes nuclear molecular imaging, which involves the imaging of molecules such as nanocarriers delivered to the body of a living multicellular organism. In this regard, scintigraphic imaging can be used for the tracking of gamma-emitting (photon) radionuclide compounds *in vivo* for diagnostic and therapeutic purposes (Boerman et al., 2000; Patel et al., 2016). The γ-ray emissions from the radionuclides can be detected by single-photon emission computed tomography (SPECT) to construct an image or be used to estimate radiation dosimetry (Jackson et al., 2020; Zhang et al., 2020).

This study utilizes gamma scintigraphic imaging to track the movements of niosomes in mice. The niosomes will first be labeled with 99mTc, then injected into tumor-bearing mice for tracking and imaging. The radioactivity in each organ indicates the presence of niosomes, which allows us to understand the biodistribution pattern and the fate of the niosomes in mice real-time.

## 2 Materials and Methods

### 2.1 Materials

D-α-tocopherol polyethylene glycol 1,000 succinate (TPGS), 4-dimethylaminopyridine (DMAP), diethylenetriamine-pentaacetic acid (DTPA) anhydride, stannous chloride dihydrate (SnCl_2_⋅H_2_O), Sorbitan monostearate (Span) 60, cholesterol, PBS, and pyridine were purchased from Sigma- Aldrich (St Louis, MO, United States). Dimethyl sulfoxide (DMSO) and BALB/c mouse serum was obtained from Fisher Scientific (Novi, MI, United States). Dialysis tubing (molecular weight cut-off 1,000 Da) was purchased from Spectrum Labs (San Francisco, CA, United States). Sodium bicarbonate (NaHCO_3_), sodium hydroxide (NaOH), and hydrochloric acid (HCl) were obtained from Systerms ChemAR (Shah Alam, Malaysia). Sephadex G-25 column was purchased from GE Healthcare Bio-Sciences Corp. (Piscataway, NJ, United States). Instant thin layer chromatography (ITLC) silica gel (SG)-coated fiber glass sheet was obtained from Agilent Technologies (Santa Clara, CA, United States). Drytec 99mTc generator (^99^Mo activity: 508.4 mCi) was purchased from GE Healthcare United Kingdom Ltd. (Little Chalfont, United Kingdom). Breast cancer 4T1 cells were obtained from ATCC, United States. Dulbecco’s modified Eagle’s medium (DMEM), fetal bovine serum (FBS), trypsin and penicillin was purchased from Gibco, United States.

### 2.2 Methods

#### 2.2.1 Preparation of Niosomes

TPGS-DTPA was synthesized and characterized following methods described previously (De Silva et al., 2019). Niosomes were prepared by simple sonication method as previously described by De Silva et al. (2019). Briefly, a mixture of Span 60, cholesterol, TPGS and synthesized TPGS-DTPA at molar ratio of 7.5:7.5:1:2 were vigorously stirred in PBS for 1 h at 60°C. Sonication was performed using a Q125 sonicator (Qsonica L.L.C, Newton, CT, United States; max 125 W) with the instrument set at 75% of its maximal capacity (measured power 94 W) for 8 min. Excess unbound materials were removed by size exclusion chromatography using Sephadex G-25.

#### 2.2.2 Particle Size and Zeta Potential Measurements

The particle size diameter (nm) and polydispersity index (PDI) of the niosomes were measured in triplicates by cumulant analysis with DLS using the Zetasizer Nano ZS (Malvern Instruments, Malvern, United Kingdom). The analysis was performed using a He-Ne laser (wavelength of 633 nm) and a detector angle of 173° (refractive index = 1.33, viscosity = 0.8872 cP) at 25°C. Samples were diluted with Milli-Q water (dilution factor 1:1,000) before analysis. Particle size diameter measurements data are expressed as mean ± SD (nm).

The zeta potential (mV) of the niosomes was measured in triplicates using Laser Doppler Electrophoresis on Zetasizer Nano ZS (Malvern Instruments). Samples were diluted with Milli-Q water (dilution factor 1:1,000) before analysis. Zeta potential measurements data are expressed as zeta potential ±SD (mV).

#### 2.2.3 Morphology of Niosomes

The niosomes were viewed using transmission electron microscope ([TEM], LIBRA 120; Carl Zeiss Meditec AG, Jena, Germany) operating at 120 kV. The niosomes were diluted with a Milli-Q water (dilution factor 1:10) before viewing. A drop of the diluted sample was then placed on a copper grid and allowed to air-dry at room temperature. Negative staining was performed with phosphotungstic acid. Any excess liquid was removed by drawing off with a piece of filter paper and left to dry in a desiccator. For viewing, the grid was mounted in the instrument and the electron images were captured under magnification of ×800.

#### 2.2.4 Radiolabeling of Niosomes

The niosomes were radiolabeled with 99mTc by surface chelation with TPGS-DTPA. 99mTc-pertechnetate solution (Na^99m^TcO_4_^−^) was obtained by elution from a sterile 99Mo/^99m^Tc generator using 0.9% w/v NaCl solution. Briefly, 1 ml of 99mTcO_4_^−^ (2 mCi/ml) was mixed with SnCl_2_.H_2_O. The pH was adjusted to pH 5 using 0.5 M NaHCO_3_ or 0.1 M HCl solution. To that mixture, 1 ml of niosomal suspension was added and left to stand at room temperature for 15 min.

#### 2.2.5 Radiolabeling Efficiency

The percentage of radiolabeling efficiency of the radiolabeled complex was measured using methods described earlier [22]. The radiolabeling efficiency was estimated with ascending ITLC using 1 × 10 cm SG-coated fiber glass sheets (Agilent Technologies, Santa Clara, CA, United States) as the stationary phase. Approximately 2–3 μl of the radiolabeled complex was applied at a point 1 cm from one end of the ITLC-SG strip and allowed to dry at room temperature. The strips were allowed to develop for 8 cm from the point of application in appropriate solvent systems. ITLC was performed using two separate mobile phases.

During the radiolabeling process, three products were generated, namely 99mTc-labeled niosomes, 99mTcO_4_^−^ and radiocolloids. Saline was used to determine the percentage of 99mTcO_4_^−^ that moved with the solvent front (R_f_ = 1.0), while radiocolloids and 99mTc-labeled niosomes remained at the origin (R_f_ = 0). A separate mobile phase containing pyridine, acetic acid, and water was used to determine the amount of radiocolloids, where radiocolloids remained at the origin (R_f_ = 0) while 99mTcO_4_^−^ and 99mTc-labeled niosomes moved to the solvent front (R_f_ = 1.0).

After developing the strips in each mobile phase, the strips were dried and cut horizontally into two equal halves and the radioactivity (counts per minute (CPM)) in each segment was determined using an automated well-type gamma ray counter (2,470 Wizard^2^ Automatic Gamma Counter; PerkinElmer Inc, Waltham, MA, United States). The percentage of radiolabeling efficiency was determined by subtracting the sum of percentage of 99mTcO_4_^−^ and radiocolloids from 100%.

#### 2.2.6 *In vitro* Stability Study of 99mTc-Labeled Niosomes

The *in vitro* stability of the 99mTc-labeled niosomes was determined by ascending ITLC-SG technique. 99mTc-labeled niosomes (0.1 ml) was incubated with 0.9% saline or mouse serum (1.9 ml) at 37°C for up to 8 h. After 0, 1, 3, 6 and 8 h of incubation, samples were drawn and ITLC was carried out to assess the percentage of dissociation or degradation of the 99mTc-labeled niosomes.

#### 2.2.7 Tumor Implantation

All animal experiments conducted were in accordance to and approved by Universiti Kebangsaan Malaysia Animal Ethics Committee (MONASH/2016/ALICE/23-MAR./731-MARCH-2016-DEC.-2018) for the purpose of control and supervision on experiments on animals. Female BALB/c mice (aged 4–6 weeks), weighing between 17 and 21 g were selected for this study. The mice were housed in a pathogen-free isolation facility under a 12 h light–dark cycle at 22–24°C and 50% humidity, and were fed *ad libitum* diet and water.

Mouse breast tumor cells 4T1 (ATCC, Manassas, VA, United States) were maintained in DMEM supplemented with 10% FBS and 100 units/mL penicillin/streptomycin. Cells were kept in a humidified atmosphere containing 5% CO_2_ at 37°C. For tumor inoculation, 4T1 cancer cells within 70–80% confluence were trypsinized, centrifuged (5 min at 320 g) and re-suspended in serum-free medium at a concentration of 1 × 10^7^ cells/ml.

The mice were inoculated subcutaneously with 4T1 cells (1 × 10^6^ cells/mouse) on the right flank. Tumor growth was monitored daily and tumor size was measured with a digital caliper. Tumor volume (V) was calculated by using longitudinal cross section (L) and transverse section (W) as follows:

$$V\left(mm^{3}\right)=W^{2}\times \frac{L}{2}$$

#### 2.2.8 Scintigraphic Imaging

Tumor-bearing BALB/c mice were used for scintigraphic imaging and biodistribution studies. When tumors reached a volume of ∼300 mm^3^, the mice (*n* = 3) were injected intravenously (tail vein) with 99mTc-labeled niosomes (200 μCi; pH adjusted to 7 with 0.5 NaHCO_3_) or 99mTcO_4_^−^ (200 µCi). At pre-determined time-points (1, 3, 6 and 8 h post injection), the mice were anesthetized with a mixture of ketamine/xylazine/Zoletil 50^®^ for the acquisition of images. The mice were placed horizontally under the collimator of a gamma camera (BHP6602 T-Quest, Hamamatsu, China) and kept under anesthesia for all the duration of the imaging. Images were acquired using a 1,024 × 1,024 matrix size at 140 keV. Scans were carried out until 200,000 counts were obtained. Interpretation of the images were done visually.

#### 2.2.9 Biodistribution Studies

Biodistribution studies were carried out with adaptions from a method by Arulsudar et al. (2003). When tumors reached a volume of ∼300 mm^3^, the mice were injected intravenously with 99mTc-labeled niosomes (200 μCi; pH adjusted to 7 with 0.5 NaHCO_3_). Mice (*n* = 5) were sacrificed at 1, 3, 6 or 8 h post-injection; blood samples were immediately obtained by cardiac puncture. Whole liver, spleen, kidney, muscle, lungs, heart, stomach, intestines and tumor were all dissected and removed, washed with 0.9% w/v saline, dried and placed in pre-weighed plastic test tubes.

To correct for physical decay and to calculate the radiopharmaceutical uptake in each organ, a standard dosage containing suitably diluted aliquots (dilution factor of 50) of the injected solution was counted simultaneously in a separate tube at each time point. The samples were counted against 1 ml suitable diluted aliquots of the injected solution. The radioactivity in each organ was counted using a well-type gamma counter and CPM values were calculated as the percentage injected dose per Gram of wet tissue. The data from the gamma counter was converted to percentage of injected dose (%ID) by dividing the CPM in each tissue or organ with the total standard dose CPM and multiplying it with 100%. The %ID/g of tissue or organ was obtained.

Formulae for calculation of %ID/g of tissue or organ are stated below:

Standard Dose(SD)=Standard count× 50(dilution factor)

Injected Dose (ID)=Standard  Dose−Tail Count

$$\%\;\mathrm{of}\;ID\;in\;tissue\;or\;organ=\frac{Count\;in\;tissue\;or\;organ}{ID}\times \;100\%$$

$$\%\;of\;ID\;per\;g\;of\;tissue\;or\;organ\left(\%\;ID/g\right)=\frac{\left[\%\;of\;injected\;dose\;in\;tissue\;or\;organ\right]}{\left[weight\;of\;tissue\;or\;organ\right]}$$

#### 2.2.10 Statistical Analysis

All data are reported as mean ± SD. The assessment of statistical significance among the results was carried out by Kruskal–Wallis followed by Dunnett’s post hoc test using GraphPad PRISM 7.0 (GraphPad Software, Inc, La Jolla, CA, United States), and *p* < 0.05 was considered statistically significant.

## 3 Results

### 3.1 Characterisation of Niosomes

**FIGURE 1:**
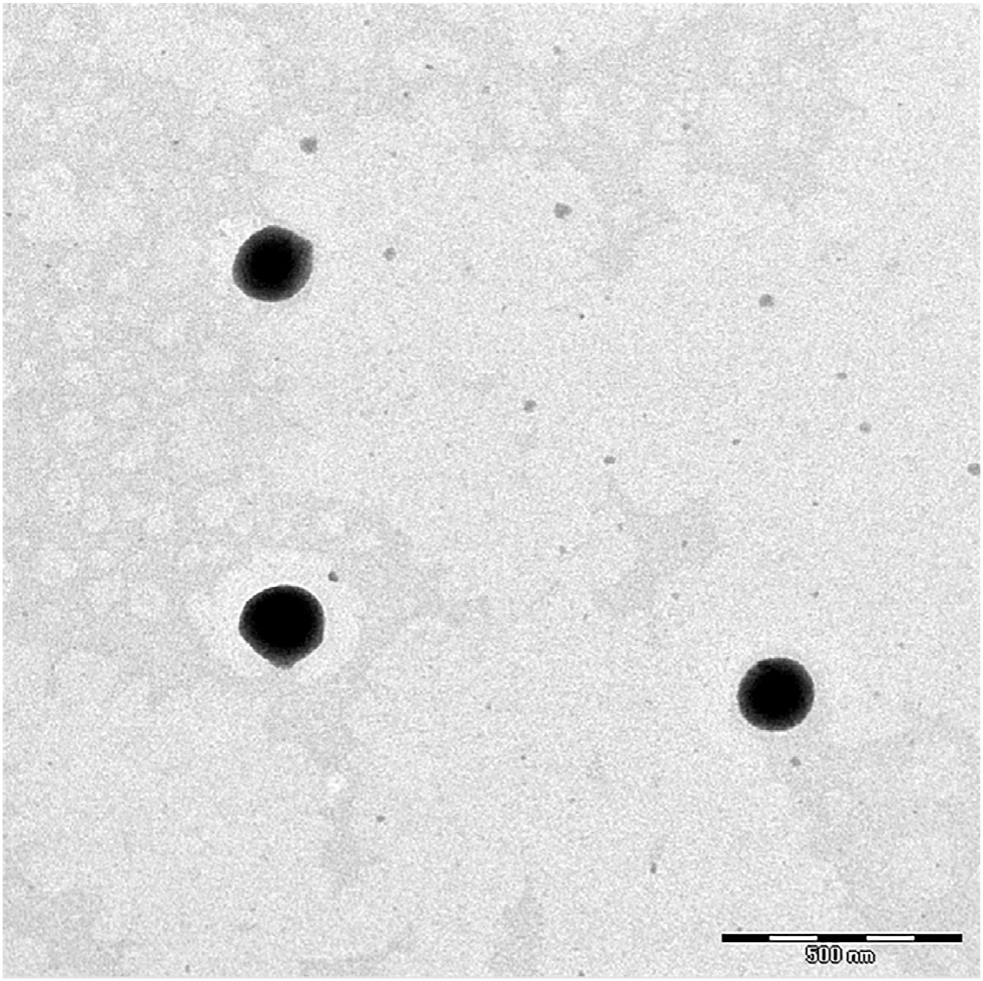
TEM micrograph of niosomes at magnification of ×800.

The formulated niosomes had an average particle size of 110.2 ± 0.7 nm with a PDI 0.229 ± 0.008, indicating good homogeneity of the dispersion. The measured zeta potential showed that the niosomes were negatively charged, with an average value of −64.8 ± 1.2 mV, indicating that the formulated dispersion is stable. The radiolabeling efficiency of the 99mTc-labeled niosomes was found to be 98.08%. Figure 1 shows the TEM image of the niosomes taken under magnification of ×800, and the size corroborates the data from zeta sizer.

### 3.2 *In vitro* Stability Study of 99mTc-Labeled Niosomes

**FIGURE 2:**
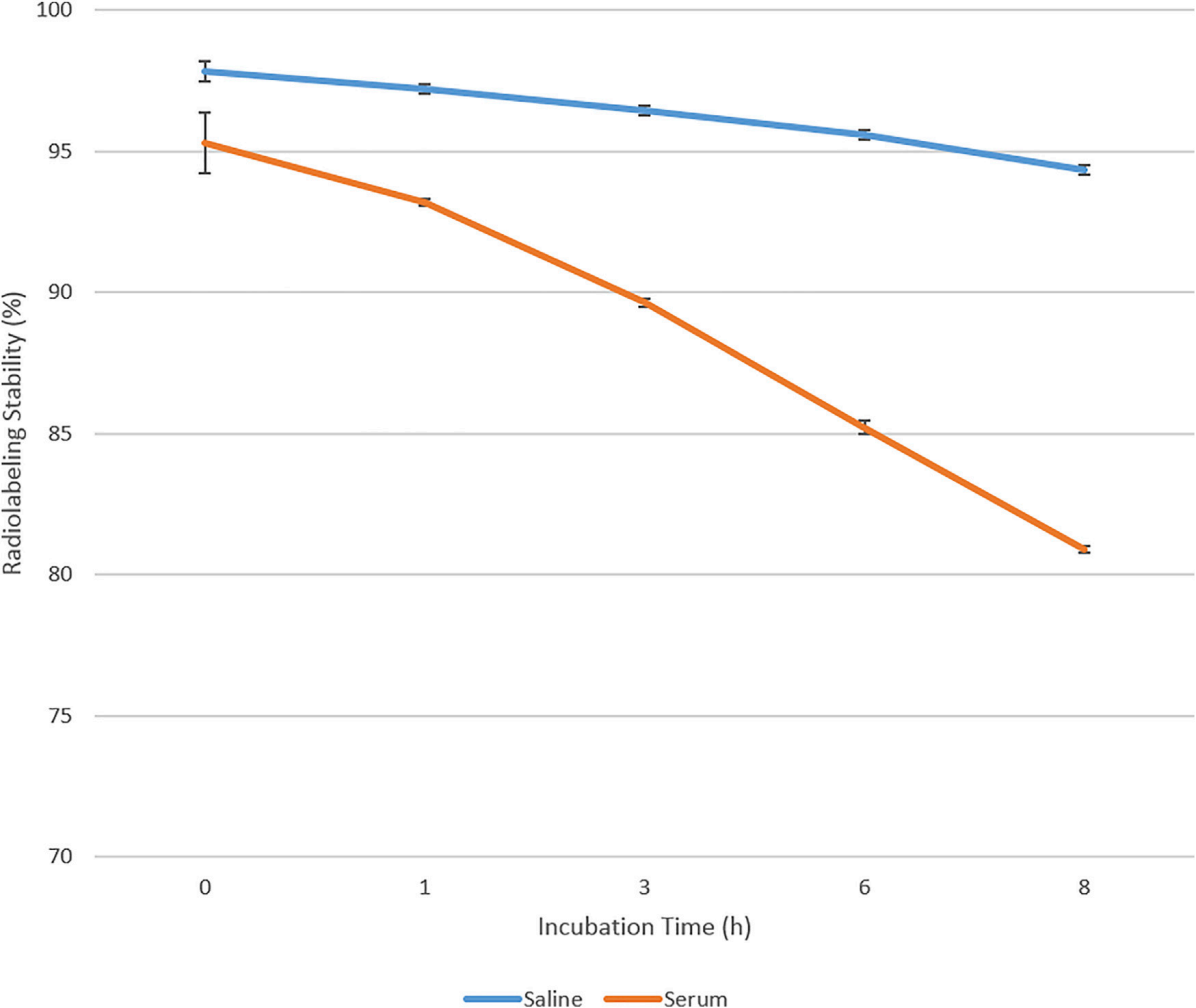
*In vitro* stability of 99mTc-labeled niosomes.

The stability of 99mTc-labeled niosomes was estimated by incubating the radiolabeled compound in saline and mouse serum at 37°C for pre-determined time intervals of up to 8 h (Figure 2). This is to mimic the condition in mice and to ensure that the radiolabeled compounds can withstand the *in vivo* condition before injection into the animals. The results revealed good stability of the 99mTc-labeled niosomes in these conditions. The 99mTc-labeled niosomes showed no significant reductions in radiolabeling efficiency with a loss of 3.50 and 14.40% of radioactivity in saline and serum respectively after 8 h, indicating that there was little dissociation of 99mTc and that the 99mTc-labeled niosomes are suitable to be used for further *in vivo* studies. The values are in agreement with values reported in the literature on *in vitro* stability (De Barros et al., 2010; Eid Moustapha et al., 2016).

### 3.3 Scintigraphic Imaging

**FIGURE 3:**
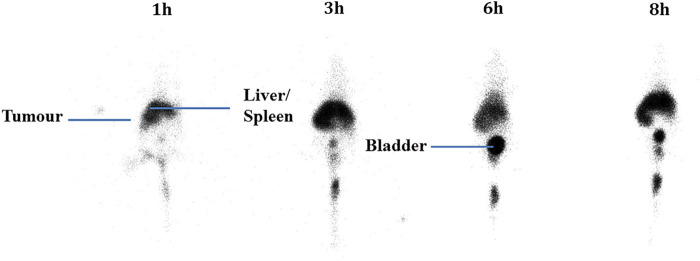
Scintigraphic images acquired from a female nude BALB/c mice at 1, 3, 6 and 8 h post-injection with 200 µCi 99mTcO_4_^-^

**FIGURE 4:**
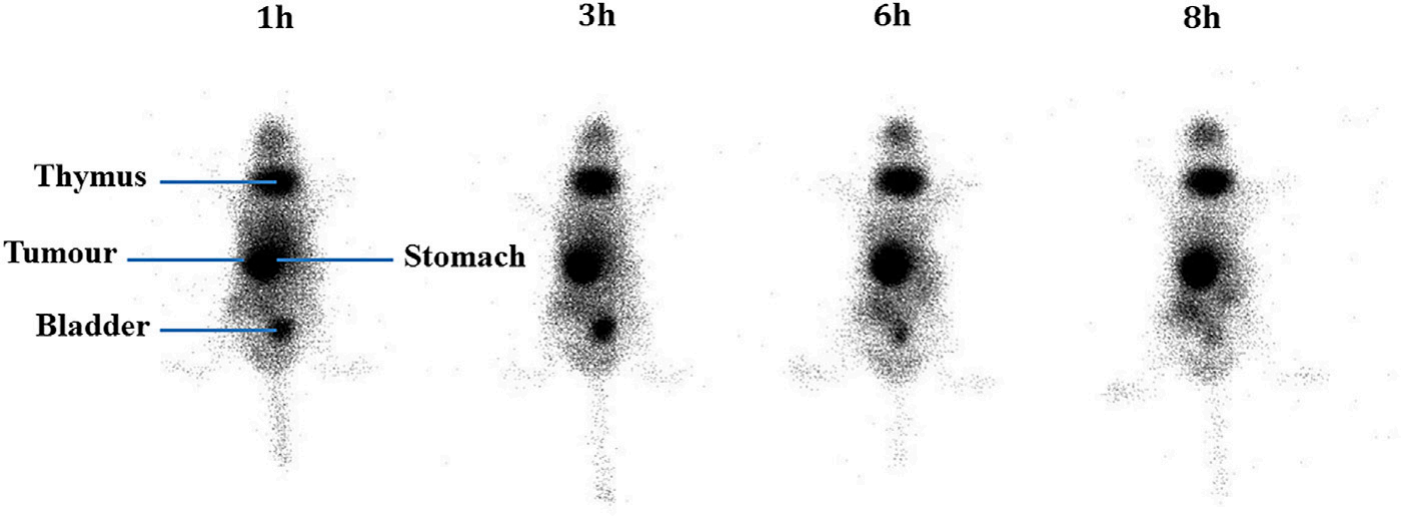
Scintigraphic images acquired from a female nude BALB/c mice at 1, 3, 6 and 8 h post-injection with 200 µCi 99mTc-labeled niosomes.

After injection into the animals, the biodistribution of free 99mTcO_4_^−^ and 99mTc-labeled niosomes in tumor-bearing mice was visualized by scintigraphic imaging. The images in Figure 3 show that 99mTcO_4_^−^ accumulated predominantly in the stomach, intestines and thymus gland. In contrast, images of 99mTc-labeled niosomes (Figure 4) showed accumulation predominantly in the liver and spleen with minimal to no radioactivity uptake in the stomach and thyroid, indicating minimal leaching of 99mTcO_4_^−^ from the radiolabeled complex *in vivo*. The differential distribution profiles of free 99mTcO_4_^−^ and 99mTc-labeled niosomes are demonstrated here and verifies that the results seen in Figure 4 are not due to detachment of free Tc.

### 3.4 Biodistribution Studies

**FIGURE 5:**
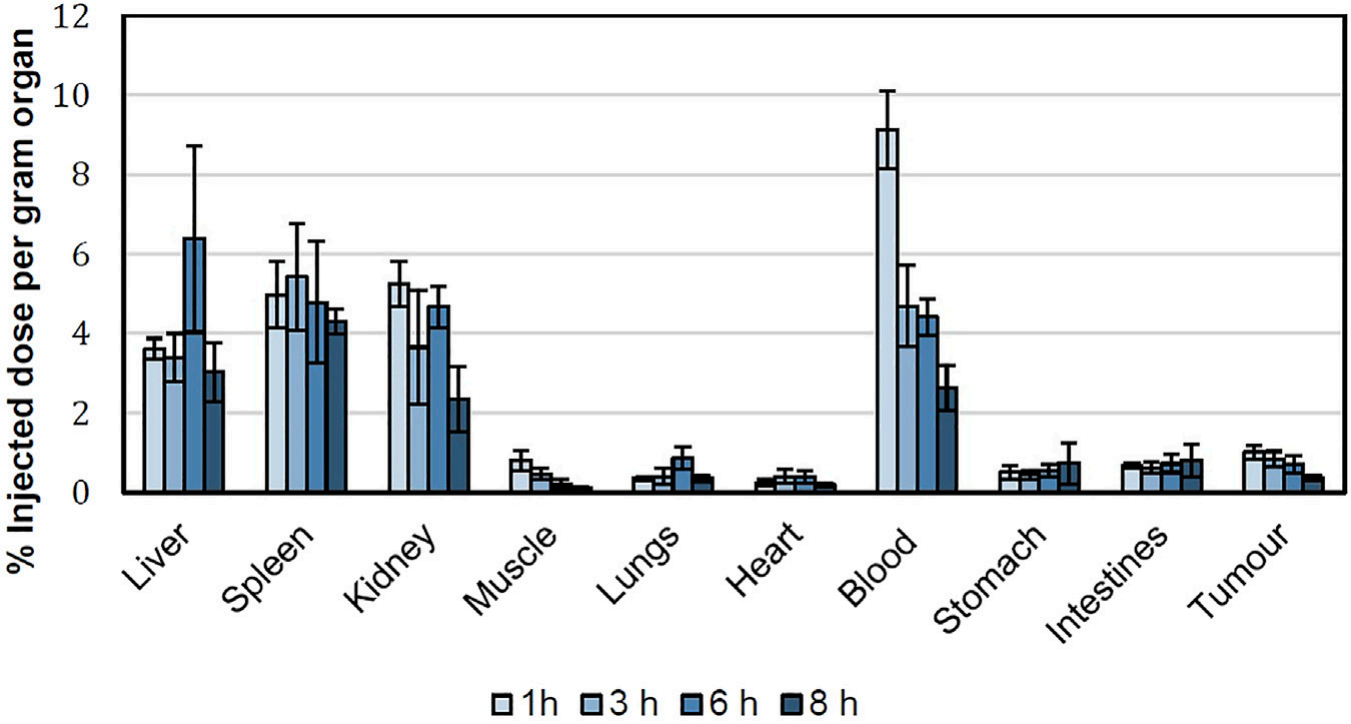
Biodistribution of 99mTc-labeled niosomes in 4T1 tumor-beating female BALB/c mice expressed as %ID/g per organ.

**FIGURE 6:**
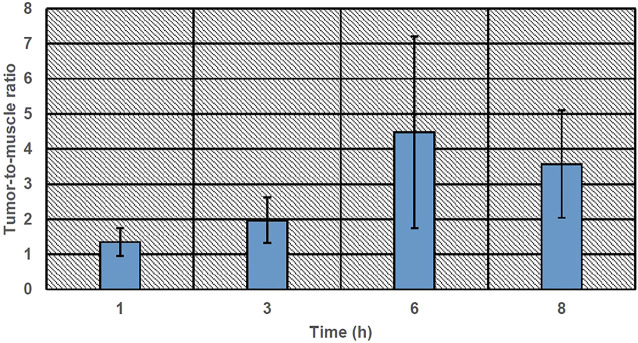
Tumor-to-muscle ratio of 99mTc-labeled niosomes.

Figure 5 shows the accumulation of 99mTc-labeled niosomes in various organs after tail vein injection in tumor-bearing mice at various time intervals. After 1 h, the niosomes were found to be located primarily in the liver and spleen with an average % ID/g of 3.61 ± 0.26 and 4.96 ± 0.84, respectively. Mean liver uptake of 99mTc-labeled niosomes dropped slightly to 3.39 ± 0.61% at 3 h, followed by an increase to 6.38 ± 2.35% at 6 h and decreased once again to 3.02 ± 0.74% at 8 h. On the other hand, the mean uptake of the spleen showed an initial increase from 4.96 ± 0.84 at 1 h to 5.42 ± 1.35% at 3 h followed by a gradual decrease to 4.78 ± 1.53 and 4.31 ± 0.32% at 6 and 8 h, respectively. Very little radioactivity was recovered in off-target organs and tissues such as the heart, lungs, stomach intestine and muscles. In the bloodstream, a peak concentration of 99mTc-labeled niosomes with a %ID/g of 9.13 ± 0.98 was observed 1 h after injection. The levels of 99mTc-labeled niosomes decreased over time, which can be attributed to the liver and spleen uptake followed by the renal elimination during the evaluated time points. The biodistribution of 99mTc-labeled niosomes in the tumor showed an initial % ID/g of 1.01 ± 0.18 at 1 h, followed by a steady decrease to 0.83 ± 0.20, 0.70 ± 0.21 and 0.35 ± 0.07 at 3, 6 and 8 h, respectively. Figure 6 shows the tumor-to-muscle ratios achieved at 1, 3, 6 and 8 h after intravenous injections of 99mTc-labeled niosomes in tumor-bearing mice. The tumor-to-muscle ratio showed an increasing trend from 1.35 ± 0.40 after 1 h, to 1.97 ± 0.65 after 3 h, followed by a maximum of 4.48 ± 2.73 at 6 h and subsequently decreased to 3.57 ± 1.53 at 8 h. However, no significant difference was detected between the time points due to high variability.

## 4 Discussion

Previous literature has shown that nanocarriers with particle sizes ranging between 100–150 nm display improved accumulation within tumors in comparison to nanocarriers with particle sizes lower than 50 nm or greater than 300 nm (Allen et al., 2006; Li et al., 2008; Ferreira et al., 2012; Fanciullino et al., 2014). Furthermore, small particle sizes confer greater stability in the circulation by the avoiding the MPS system (Fanciullino et al., 2014). The absence of microvasculature occlusion or pulmonary embolism from the systemic administration of the formulated 99mTc-labaled niosomes supports that the delivery system possesses suitable physical characteristics for *in vivo* use. The zeta potential obtained indicates strong repellant forces on the surface of the niosomes that can prevent aggregation (Patel et al., 2016).

TPGS is a non-ionic, amphiphilic molecule consisting of a lipophilic non-polar (water-insoluble) head and a hydrophilic (water-soluble) PEG tail and is formed through the esterification of D-alpha-tocopheryl acid succinate and PEG 1000 (Huang et al., 2011; Guo et al., 2013; Muthu et al., 2015; Patel et al., 2016). In an aqueous environment, the hydrophilic PEG tail becomes saturated with water molecules, thereby increasing the stability and increasing steric hindrance of TPGS coated structures (Muthu et al., 2015). It has also been reported that the size of niosomes can be reduced through the incorporation of TPGS, resulting in reduced bilayer defects in the niosomes, thereby improving the lateral packing of the acyl chains of the surfactant in the bilayer (Garbuzenko et al., 2005). In stability study, niosomes with TPGS remained stable up to 60 days as changes in size, polydispersity index and zeta potential were less than 17% (Maniam et al., 2021).

The niosomes were radiolabeled by direct radiolabeling through the reduction of 99mTcO_4_^−^ in the presence of SnCl_2_.H_2_O. In comparison, a study by Korkmaz et al. (2006) incorporated 99mTc-DTPA into the niosome by thin film evaporation (Uchegbu, 2000). While they were able to similarly achieve high radiolabeling efficiency, the method involves higher radiation exposure to the personnel, as the niosomes are radiolabeled during the start of the manufacturing process. Additionally, the short half-life of 99mTc would require immediate administration into a patient after synthesis, and requiring substantial amount of work in a limited period of time. Scintigraphic imaging has been widely used to provide information on whole body distribution of radiolabeled compounds at different time intervals in a live animal or human subject (Bao et al., 2004; Monteiro et al., 2018). Figure 3 shows a series of anterior whole-body images of the distribution of 99mTcO_4_^−^ in tumor-bearing mice captured at 1, 3, 6, and 8 h post-injection. The images indicate that 99mTcO_4_^−^ predominantly accumulated in the thymus gland and digestive system, which is in good agreement with previously reported data on the distribution of 99mTcO_4_^−^ *in vivo* (Valenca et al., 2005; Chacon et al., 2007). This accumulation occurs as the thymus gland and stomach are the main elimination organs of 99mTcO_4_^−^ (Ferreira et al., 2015; Silva et al., 2016). In comparison, in Figure 4, the images of the mouse injected with 99mTc-labeled niosomes showed minimal radioactivity in the thymus gland and digestive system, indicating minimal dissociation and degradation of the radiolabeled complex *in vivo.* The high accumulation of 99mTc-labeled niosomes in the liver and spleen at later time points can be attributed to the natural mechanism of vesicular clearance (Arulsudar et al., 2004; Gharepapagh et al., 2021). The planar images obtained from scintigraphic imaging provides qualitative information, as the activity in certain organs are seen to partially overlap (Fragogeorgi et al., 2014). For example, images of the lungs/liver, liver/spleen and kidneys/intestines could be difficult to differentiate in the images. This data is therefore supplemented with quantification study.

Quantification of radioactivity in isolated organs and tissues provides an accurate and thorough understanding of the biodistribution of 99mTc-labeled niosomes. The results were in agreement with the biodistribution profiles seen in the scintigraphic images. Upon entering the circulation, there was considerable radioactivity (median >3 %ID/g) in the clearance organs (liver, spleen and kidney). Vesicular systems are typically eliminated via phagocytosis, primarily by macrophages from the liver and spleen and consequently taken up by the reticuloendothelial system (RES). This effect has been similarly observed in previous studies with other radiolabeled niosomes (Almasi et al., 2018). In our study, the high %-ID/g observed in the liver and spleen may be attributed to the interactions between the negatively charged groups on the surface of the niosomes and the cell surface proteins (Arulsudar et al., 2004). Juliano et al. (1975) and Dobrovolskaia et al. (2008) reported that negatively charged nanoparticles exhibit higher macrophage uptake in comparison to positively charged and neutral nanoparticles as a result of their tendency to coalesce in the presence of proteins and calcium ion in blood plasma (Juliano et al., 1975; Dobrovolskaia et al., 2008). However, this phenomenon was unavoidable in our formulation as the negative charge on the surface of our nanocarrier arises from the DTPA molecule, which is required for labeling 99mTc to the niosomes.

The ideal biodistribution pattern of 99mTc-labeled niosomes would include little to no radioactivity in the thyroid gland, stomach and kidneys; indicating that the reduced 99mTc is tightly bound to the niosomes. Low radioactivity was detected in the stomach and intestines throughout the study with a maximum average %ID/g of 0.72 ± 0.53 and 0.80 ± 0.41, respectively at the 8 h time-point. The presence of a detectable amount of radioactivity noted in the stomach could be attributed to the self-grooming phenomenon that typically occurs in mice (Santini et al., 2017). The radioactivity found in the kidney following intravenous administration suggests that the 99mTc-labeled niosomes are excreted through the renal route upon splenic and hepatic metabolism (Kamal et al., 2018).

A good nanocarrier for anti-cancer drug delivery should possess a high capacity to target tumor tissues with minimal to low accumulation in off-target tissues to maximize tumor uptake and minimize toxicity to healthy tissues. In the tumor, the radioactivity was around 1 %ID/g, which was comparable to that of previously published studies (Richardson et al., 1977; Oku et al., 1993; Arulsudar et al., 2004; Fragogeorgi et al., 2014). While radioactivity at the tumor site compared to the total body activity was considerably low, there was a significant ratio of 99mTc-labeled niosomes uptake in the tumor in comparison to the muscle. Accumulation of radioactivity is affected by the route of administration (Bahadori et al., 2021), where nearly perfect tumor retention was observed when radiolabelled nanoparticles are injected intratumorally (Moeendarbari et al., 2016). In this study, niosomes were injected intravenously, hence a lower overall accumulation should be expected. In fact, literature has reported that a tumor-to-muscle ratios of larger than 1.5 signifies that there is a 50% greater capture of the compound in the tumor tissue (Phillips et al., 1999; De Barros et al., 2010), which validates our results of tumor accumulation following intravenous injection. Figure 6 shows that 99mTc-labeled niosomes had a tumor-to-muscle ratio of above 1.5 after 3 h, indicating the ability of the niosomes to accumulate within the tumor microenvironment through EPR effect. Though no significant difference can be observed between the time points, the tumor-to-muscle ratios are above 1.5 after 1 h, showing preferential tumor accumulation. The EPR effect has been a leading factor in influencing nanocarrier formulation for anti-cancer therapy due to its proven efficacy in animal models. However, the heterogeneity of the EPR response in humans has raised questions regarding the universal validity of EPR among researchers (Danhier et al., 2016). A study with radiolabeled liposomes showed positive correlation between neoangiogenesis and EPR effect while interstitial fluid pressure at tumor microenvironment had little effect (Børresen et al., 2020). Having said that, there have been many successful developments of nanomedicines based around the EPR effect with excellent treatment efficacy over the years. A phase II trial of PEGlyated liposomal doxorubicin (DOXIL^®^/CAELYX^®^) showed that IV infusion of the nanoformulation resulted in anti-tumor activity with improved toxicity profile compared to standard IV bolus of doxorubicin in patients with soft tissue sarcoma, indicating tumor specificity (Judson et al., 2001). Recent studies have also found that the concurrent administration of vascular modulating agent can help overcome this through the selective passive opening of the defective endothelial cell gaps in tumor tissues (Nakamura et al., 2015).

The limitation of the current study includes the use of animal tumor model 4T1 cells, in which all the data presented are on mice tumor. Future work could explore the use of human tumor cell lines implanted on nude mice. This would provide a tumor environment closer to human and generate added information prior to engaging in larger animal models or human studies. We also recognize that gamma scintigraphy is not the latest technology in animal imaging. More advanced imaging techniques, such a positron emitting tomography (PET) could produce images with higher resolution, but also at a higher cost. Moreover, the common radionuclide used in PET, Fluorine-18, has an extremely short half-life of 109.8 min, which is not suitable for biodistribution studies involving longer time points. Thus, gamma scintigraphy presents as a cheaper and more accesible option, with its associated radionuclide 99mTc of 6 h half-life, making it a more suitable technique for our study. By utilizing this non-invasive imaging technique, we were able to avoid the unnecessary use of a large animal sample size before proceeding to a more comprehensive and invasive biodistribution study, where animals will be required to be sacrificed at each time-point (Bao et al., 2004).

## 5 Conclusion

In conclusion, biodistribution study of nanocarriers, such as niosomes, can be carried out non-invasively using scintigraphic imaging. We demonstrated successful prototype of niosomes conjugated with 99mTc through a simple and fast 1-step method. Based on the physical characteristics and stability features, our results indicate the feasibility of the developed niosomes for *in vivo* administration. With the high tumor to muscle uptake, the niosomes can act as a potential delivery system for improved tumor uptake of anti-cancer drugs. While controversy surrounds the validity of the EPR effect in humans, we maintain the belief that PEGylated niosome at ∼110 nm is a suitable candidate to achieve effective tumor penetration via the EPR effect. The niosomes formulated in this study offers promising results as a proof-of-concept and as a prototype for future development of niosomes for passive retention applications. Further enhancement can be achieved to augment the EPR effect such as alterations in blood flow and vascular permeability. The niosomes can be further designed to conjugate active targeting ligands to its surface, to improve the targeting ability and achieve even higher niosomes accumulation within the tumor microenvironment. This can then be used to encapsulate anticancer compounds for breast cancer targeting to achieve better treatment outcomes.

## Data Availability

The original contributions presented in the study are included in the article/Supplementary Material, further inquiries can be directed to the corresponding author.
